# Antecedents of use of e-health services in Central Eastern Europe: a qualitative comparative analysis

**DOI:** 10.1186/s12913-020-5034-9

**Published:** 2020-03-04

**Authors:** Marek Ćwiklicki, Francesco Schiavone, Jacek Klich, Kamila Pilch

**Affiliations:** 1 Department of Management of Public Organisations, Cracow University of Economics, ul. Rakowicka 27, 31-510 Cracow, Poland; 2 Department of Management Studies & Quantitative Methods, University of Naples Parthenope, Via Generale Parisi 13, 80132 Naples, Italy; 3 Department of Strategy and Management, Paris School of Business, 59 Rue Nationale, 75013 Paris, France

**Keywords:** E-health, Services, Policy, Central Eastern Europe, QCA

## Abstract

**Background:**

The objective of this paper is to identify the key conditions that positively affect the use of e-health services in Central Eastern Europe (CEE) countries. CEE countries after the political and economic transformation in 1989/90 implemented slightly different national health care models. The research question of the study is: how do the various institutional conditions at the national level affect the use of e-health services in CEE countries?

**Methods:**

The e-health description was derived from papers indexed in Web of Science and Scopus. The data for computation were collected from the 2015 global survey by the WHO Global Observatory for eHealth. We used a narrative literature review in order to identify key terms associated to e-health and conditions for the implementation of e-health services. The search terms were “e-health” and “*” where * was particular thematic section of e-health according to WHO GOeH. The inclusion criterion was relevance of the paper to e-health and searched phase. Eligibility criteria for countries for being described as CEE countries: Estonia, Lithuania, Latvia, Poland, Hungary, Romania, Bulgaria, Czech Republic, Slovenia, and Croatia (we omitted Slovakia from the analysis because this country was not covered by the WHO Survey). We applied qualitative comparative analysis (QCA) to analyse the necessary order of conditions. The dependent variable of the study is the national rate of use of e-health services.

**Results:**

QCA shows that legal medical jurisdiction, teleprogramme and electronic health records supplemented by adequate training constitute critical conditions to achieve success in e-health implementation.

**Conclusions:**

We conclude that the more formalised a framework for e-health service delivery is, the more likely it will be used. Therefore formalisation fosters the diffusion, dissemination and implementation of e-health solutions in this area. Formalisation must be accompanied by tailored training for health care professionals and patients. Our analyses are related only to the paths of e-health implementation in CEE countries thus consequently the findings and conclusions cannot be directly applied to other countries. The limitations of this study are related the absence of a broader context of e-health development, including the development of ICT infrastructure and ICT literacy.

## Background

Digital transformation is one of the main current challenges for companies and institutions in every business sector [1, 2]. For a long time, healthcare players have been trying to digitalise their processes, practices and services [3, 4]. Health IT (HIT) offers crucial benefits to companies and institutions in terms of both quality of care (e.g., patient satisfaction and safety) and efficiency/financial performance (e.g., costs and value added) [3].

To date, one of the main paths crossed by healthcare organisations in implementing digital transformation is the so-called e-health [5]. Within ICT for health, the e-health concept began to appear in the literature in 1999 as an evolution of the telehealth domain [6]. Scholars have greatly explored this topic. E-health refers to all of the “health services and information delivered or enhanced through the Internet and related technologies” [7]. Consequently, ICT is seen as the only channel through which various e-health programmes and initiatives can be provided thus can be seen as a main determinant of e-health. The components of e-health are electronic health records (EHRs), health information, clinical decision support systems, and physician order entry [6]. Since e-health is considered here broadly, as an umbrella term, it covers also subsets like telehealth, telemedicine, and m-health. E-health solutions require the involvement and integration of more knowledge and scientific domains, such as medical informatics, public health, and business [7]. However as Scott and Mars notes: “We have still not agreed upon a universal, standard definition of eHealth, and related terms” [8].

E-health services can be differentiated in terms of technologies, functions, and fields of application [6]. The main e-health services offered to patients can be classified into five categories [9]: 1) Education services, by which institutions can use digital technologies to improve consumers’ access to health-related information; 2) E-behaviour change services, by which online support groups and collaborative communities can promote novel and healthier lifestyles among patients; 3) Self-monitoring and disease management, by which a chronic patient can track via a smartphone his/her own health status without professional support; 4) E-treatment adherence services, by which health professionals can send patients more reminders, at different moments and to different devices to increase their treatment adherence, using telemedicine; and 5) E-surveillance services, by which doctors can implement via EHR systematic and ongoing health assessment (data collection, analysis, interpretation, and dissemination of findings) of the conditions of chronic patients using electronic medical records. As indicated earlier, we consider telemedicine - just mentioned above - as an important, growing in value and numbers, subset of e-health. The widespread use of smartphones in people’s daily lives has also boosted the rise of mobile health (or m-health) which is treated as another subset of e-health. This is a fast-growing new market and technological niche born in 2003 just after e-health began [6]. The WHO Global Observatory for eHealth (WHO GOE) defines mobile health as a “medical and public health practice supported by mobile devices, such as mobile phones, patient monitoring devices, personal digital assistants (PDAs), and other wireless devices” [10]. A recent example of the successful use of m-health apps was the collection and use of patient-generated data for supporting diabetes self-management [11]. Recent study finds that “mobile health applications has a positive impact on health-related behaviors and clinical health outcomes” [12].

Despite these evident benefits, the introduction of digital services in healthcare can also generate problems, risks and drawbacks. For instance, e-health services can lead to a digital divide among the various segments of the population since some people with low socioeconomic status are less likely to know about and use these services [13]. Privacy and the use of patient data by institutions and companies are other critical issues for all the various domains of e-health [14].

**Fig. 1:**
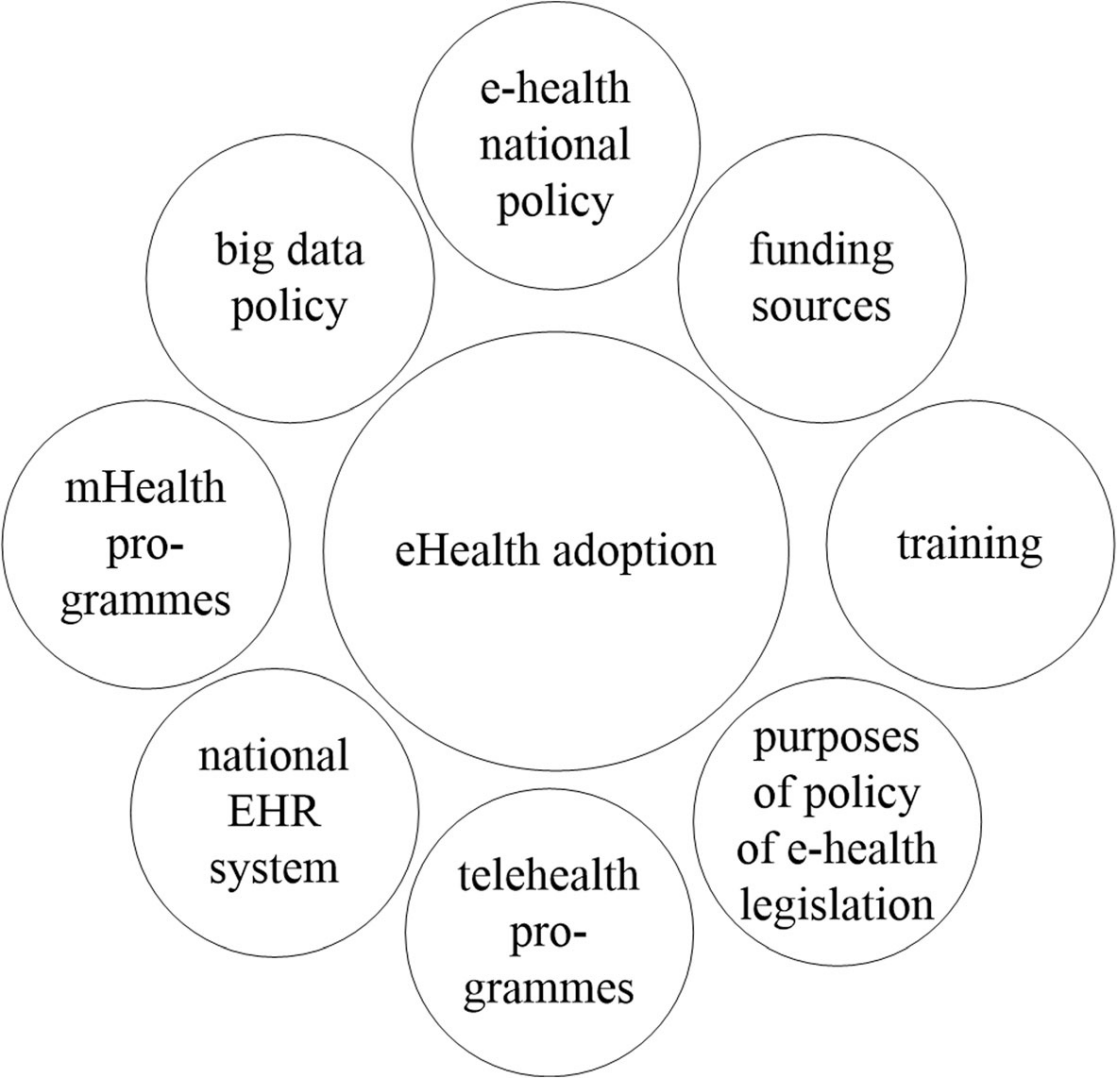
The thematic sections of e-health. Own elaboration based on [15]

A group of experts in digital health and staff members of the WHO [15] identified 8 antecedents of e-health adoption (Fig. 1), which are reviewed in detail in the next section.

The presence of elements in each of these 8 areas can be associated with successful e-health diffusion and implementation. We use the above framework to bring up particular issues and to critically examine them in light of the narrative literature review.

CEE countries form an important sub-continent for health and industrial policies. The experiences of CEE countries have greatly contributed to transitology (the process of democratisation) [16, 17]. Despite the common heritage of Semashko’s healthcare model (named after the first Minister of Health in the USSR and also called the budget model), which is) highly centralised and based on public financing and service delivery [17–19], after the 1989/90 political and economic transformation, CEE countries implemented slightly different national healthcare models. The latest developments of healthcare systems in these countries remain relatively poorly researched. This article aims at filling this gap at least partially. The objective of this paper is to identify the key conditions that positively affect the implementation of e-health services within this European sub-continent. Thus, the research question of the present paper is the following: what conditions are necessary and sufficient for achieving an effective use of e-health services in CEE countries?

## Methods

We performed a narrative review because its main purpose was to identify and map the available evidence [20]. We searched for e-health policy components in Web of Science, Scopus and Google Scholar. The review was performed in September 2018. Our search strategy used including the search terms inputted into the databases searched by adding the following explanation: The search string was {“e-health” and “*”} where * was particular thematic section of e-health according to WHO GOeH, e.g., e-health and m-health and so on. The searched areas in databases were: the paper’s title, abstract, and keywords, as well as date (as we wanted to refer to recent literature). The inclusion criteria were: relevance of the paper to e-health and searched phase (fitting into discussed thematic section and refer to CEE), publication date (novelty), source type (reviewed papers). We selected all the papers satisfying all these criteria.

We notice that the WHO GOE covers the most important e-health dimensions with strong reference to policy and strategy. However, the national framework is also a crucial element for spreading the use of e-health.

The implementation of strategies referring to new areas of public service provision requires the adoption of appropriate methodological rigour that fits in the third generation of research on public policy introduction [21]. The qualitative comparative analysis (QCA) method, developed by Ragin, uses Boolean algebra to analyse real patterns and logical formulae. It allows for the determination of logical conclusions from the dataset. In this method, cases are presented as a set of factors and conditions influencing a given effect. QCA is focused on both the case and the variables [22].

Analyses carried out with the use of QCA rely on the identification and calculation of all combinations of the variables occurring in the dataset. A condition is considered necessary if it must be present to achieve a given result and sufficient if it creates the result itself [23]. As Ragin wrote, no single causal condition may be either necessary or sufficient for the outcome in question; instead, only combinations of conditions may be found to be sufficient [22]. QCA minimises the configuration and strictly applies the Mill method, according to which if two configurations differ with respect to only one condition but have the same result, this condition is irrelevant and can be eliminated [22].

Our goals were to identify the key conditions to achieve success in e-health implementation at the national level. At the same time, we wanted to identify which ones are necessary and which are sufficient. Therefore, we first identified (based on the narrative literature review) the outcomes and the conditions to be assessed. The proposed approach and method have been successfully used in other health care studies in which the goal was to identify necessary and sufficient factors (e.g., [24–26]). The QCA method suggests theoretically plausible conditions that with further development could help meet the e-health implementation challenges.

CEE countries are often bypassed in comparative studies into e-health partly because they are behind the developed democracies with respect to ICT [27, 28]; thus, a concise picture of the most recent developments in the analysed countries seems to be appropriate. Therefore, we present a general description of these countries and the e-health solutions available in each. We present the following countries: Slovakia, Estonia, Lithuania, Czech Republic, Bulgaria, Hungary, and Poland. Next, we discuss all CEE countries in contrast to all EU member states based on composite indicators.

The overall picture of e-health solutions in operation in the analysed countries is far from complete due to the lack of information, on the one hand, and the dynamics of the process itself, on the other. Below, we present a brief description of the e-health solutions in those CEE countries based on the official reports, announcements, etc. available on national ministry and medical business websites.

As research material, we used data from the third global survey on e-health conducted by the WHO GOE. We focused on the data for 10 Central Eastern European countries (Estonia, Lithuania, Latvia, Poland, Hungary, Romania, Bulgaria, Czech Republic, Slovenia, and Croatia). Because Slovakia was not covered by the WHO survey, we omitted this state from analysis. fsQCA 3.0 software was used to carry out the analysis [29].

The overall research procedure consists of three steps.

1. Measuring outcomes: First, we established the measure for the outcomes. We associated the outcome with the successful delivery of e-health services, which we understand as a high percentage of e-health service use by patients. We used as the measure for outcomes the percentage of use of two e-health services included in the European Life Quality Survey in 2016: prescriptions ordered online or by telephone and medical consultations held online or by telephone [30]. We assumed that if the percentage of prescriptions ordered online or by telephone is greater than or equal to 20 and the percentage of medical consultations held online or by telephone is greater than or equal to 15, this indicates successful implementation of e-health policies.
2. Identifying and assessing conditions: The next step is to identify relevant causal conditions derived from the WHO Global Observatory for eHealth survey [31, 32]. Based on the narrative literature review in one of the previous sections, we identified the conditions contributing to the use of e-health services in the CEE countries. To avoid redundancy in the data, we chose the conditions with reference to policy and strategy. We decide to exclude certain conditions from our analysis. As our scope of interest concerns the CEE countries, which are mono-cultural and mainly mono-ethnic, we did not consider the issue of *multilingualism in e-health*. Because the *use of e-learning in health sciences* is covered in the broader concept of capacity building, we do not include it in further examination. According to literature, it is relevant at the individual level, as in doctor-patient interaction. Therefore, it is more relevant for patients and health organisations than for national regulation or policy. The next condition is *big data*. Although it is an important issue, as suggested in the literature, it is loosely connected to patients’ direct use and is more appropriate for e-health governance at the national level. Moreover, the collected data cover this issue to a lesser degree. In the survey, particular e-health programmes were assessed with respect to different system levels (local, intermediate (regional), national, international) and programme types (informal, pilot, established). We chose pilot or established programmes at the national level.
3. Finally, we identified the combination of conditions for the use of e-health. The QCA method enables the study of the most significant similarities and differences among the analysed cases and indicates one or more combinations of conditions that may be equally effective. Using the numerical values for the outcomes and conditions for each of the 10 cases, a truth table was produced. This table displays all of the possible combinations of conditions leading to the outcome.

## Results

In our analysis, there are eight the building blocks of e-health adoption. The first block is the e-health foundations, which are (a) national policies/strategies, (b) funding sources for e-health, (c) multilingualism in e-health, and (d) e-health capacity building.

### National e-health policies

National policies are important drivers for e-health solutions. At the operational level, the analysis of implementation factors of e-health service adoption has been described in recent works [33]. The analysis of e-health systems showed that the most important issue was their workability, with less emphasis on efficiency [34]. Workability can be assured by a proper legal framework, among other aspects. However, e-health legislation is considered a new issue. Mars and Scott assess legislation in some developed countries as ad hoc patchwork [35]. The authors observe that “information on country-level e-health policies and strategies is not readily available” [35]. An inadequate or fragmented legal framework was mentioned in the e-health Action Plan 2012–2020 among the barriers to the deployment of e-health. Mars and Scott state that e-health policy is fragmented and encompasses different, even separate, yet related strategies, programmes, and national action plans.

In response to social and economic changes, the WHO formulated the guidelines for implementing national e-health strategies [27], and the European Union accepted a respective action plan for the period 2012–2020 [36]. E-health strategies are diffusing. The WHO GOE’s report [15] shows that 58% of surveyed countries have an e-health strategy and that 66% of them have a national health information system policy or strategy. Furthermore, particular EU countries have started to introduce national e-health strategies, which we discuss in the next paper’s section with reference to CEE countries. We can conclude that the more a framework for e-health service delivery is formalised, the more likely it is used. Therefore, we assume that formalisation fosters the diffusion, dissemination and implementation of e-health solutions in this area. We look at the broader context in the model of innovation diffusion in health service organisations [37].

To be more accurate, we refer to external policies and incentives [38] at the national level. We claim that the success of the implementation of e-health services depends on the degree of intensification of established programmes, legal frameworks, state-run policies, etc., which can be named the formalisation of business rules for service delivery or the introduced strategies, regulations and programmes. “National projects” are understood as initiatives undertaken by the Ministry of Health (or/and other central bodies) and implemented nationwide. Of course, there could be other interesting ventures/projects initiated on a small, local scale. However, to obtain a full, concise and heterogeneous picture, we decided to examine “national projects”.

### Funding sources for e-health

Reports of financing e-health show that the integration of funding sources is the most significant obstacle [39]. It is suggested that the best way is that of mixed arrangements [39]. This is also observed in a recent study on financial resources and the development and implementation of e-health infrastructure and applications [40]. An analysis of e-health policies shows that public-private partnerships are encouraged [41]. Moreover, healthcare organisations should invest in e-health. Nevertheless, the healthcare systems in CEE were developed according to Semashko’s model. According to the model, healthcare was financed via state budgets, with citizens having free access to health services [18, 19].

### Multilingualism in e-health

According to the WHO GOE, “a national multilingualism policy or strategy promotes linguistic diversity and cultural identity and reflects a government’s commitment to inclusion of linguistic minorities in the country” [15]. This issue in scholarship is not undertaken as a key area of introducing e-health. The research refers to multicultural countries, especially those receiving large immigrant populations with limited national language proficiency [42, 43].

### E-health capacity building

The fourth topic influencing the adoption of e-health is generally described as capacity building. By this expression, pre- and in-service training in e-health are understood. The first refers to health science students, and the second refers to health professionals. The issue of possessing the skills with which to operate e-health technology is essential in spreading the use of e-health. As research demonstrates, “educating health care professionals with the necessary skill training in e-health care will improve service delivery” [44]. Findings also refer to the education process.

The second building block of e-health is the legal framework [41]. It is argued that “more specific e-health law would help clarify roles, rights and responsibilities of the various stakeholders” [45]. As a lack of trust is perceived to be one of the main obstacles to the implementation of e-health solutions, Geissbuhler states, “Trust amongst stakeholders can be strengthened through consensual formalization of rules into a specific law” [45]. Therefore, we can conclude that the more differentiated the purposes of legislation policy, the better it is for e-health adoption.

The telehealth block covers five national programmes: teleradiology, teledermatology, telepathology, telepsychiatry, and remote patient monitoring. A study on telehealth adoption among US hospitals showed that the rate of hospital telehealth is associated with differences in state policy [1]. The most common aspect of research is on an organisational level, i.e., telehealth adoption in hospitals [46] or among individuals from specific groups, such as older people [47–49]. As telehealth is considered at an individual level (clinician to clinician, clinician to patient), it is also connected with the health professional workforce and – to some extend – with electronic health records [50]. Detailed studies have been conducted on the electronic health communication between physicians and patients [51]. The bottom-up approach, i.e., a patient’s perspective, has also been considered in the study of factors influencing the adoption of e-health in developing countries [52].

The fourth block is electronic health records (EHRs). It is divided into four parts. The first constitutes the national EHR system and legislation governing its use. The other parts are health facilities with EHRs, the existence of the domain electronic system (e.g., laboratory, pathology), and ICT-assisted functions, such as electronic billing and supply. EHRs cover different information systems that have various scopes supporting single functions, activities for particular organisations, more interoperability of the solutions used at the national level, etc. [53]. Research into the community level of EHR use has proved that data collection and presentation in this area should be standardised [54].

According to a recent study, the use of e-learning in health education (fifth building block) is still not fully recognised and requires a model for evaluating learning in e-health [55]. Nevertheless, a systematic review of the effectiveness of e-learning showed that this form of education was evaluated slightly higher than traditional education means but with some limitations [56].

M-health programmes (sixth building block) are divided into three groups: accessing/providing health services (6), accessing/providing health information (5), and collecting health information (3).

An analysis of the previous WHO survey showed that m-health is “characterised by small-scale pilot projects that address single issues in information sharing and access” [10]. The narrative literature review suggests that m-health projects lack a complex design targeted at point solutions [57].

Social media (seventh building block) cover three issues. The first one concerns policy/strategy regulating the use of social media in the health domain by governmental organisations. The next deals with healthcare organisations using social media. The last one refers to the use of social media by individuals and communities. Literature about using social media in the e-health context suggests that the motive behind such use is that of self-care and self-management [58] or patient empowerment [59]. It also noticed that social media are reshaping the way in which doctors and patients interact [60].

The eighth and last building block is big data. In the WHO survey, this issue is covered by two related topics dealing with governing the use of big data in the health sector and by private companies. Big data are perceived to be one of the most important issues that must be considered in e-health solutions [61]. Studies show a broad spectrum of potential utilisation, yet there are still some serious challenges to overcome in the area of big data analytics [62].

Among the most recent changes in the e-health systems implemented in Estonia and Lithuania is a special portal dedicated to patients that allows them to monitor their medical data and appoint representatives and health information systems (with electronic health records), allowing doctors to obtain quick access to a patient’s complete health information. As natural consequences of these two, e-prescription was initialised, accompanied by digital registration and electronic medical certificates.

In the Czech Republic, legal regulations on patients’ medical records are being formulated, and only e-prescription is operational but only on a limited scale [63]. Ordering prescriptions by telephone (or to be exact: by emailing a primary physician) is considered as (relatively) simple procedure, very appreciated by patients. Ordering prescriptions by email is well developed only in Estonia. Such a system is in its infancy in Poland in 2019.

In Bulgaria, measures such as electronic consultation requests, e-prescriptions, and electronic health registers have not yet been achieved, although the “Strategy for the Implementation of e-Healthcare in Bulgaria” has been adopted [64]. In Hungary, the implementation of the e-health system is progressing. Primary care physicians operating in the public sector as well as pharmacies had to register by 1 November 2017 in the national electronic health records system, while private primary health practices were supposed to do so by 1 November 2018 [63].

In Poland, e-health solutions have been a policy priority for over a decade, but progress has been slow and patient health books are still kept in paper form. E-prescriptions and e-referrals are still on the initial steps of development since full implementation of these two programmes has been postponed a number of times (currently until 2020–21). Furthermore, the implementation of e-solutions is more advanced in the hospital sector, especially in the largest hospitals, than in ambulatory care. E-doctor sick-leave certificates have been launched recently. As a result, the functionality of e-health in Poland has been questioned [65].

To summarise: Comparative studies on e-health in CEE countries are rare. The scope of e-health-related conditions is diversified in CEE countries, both within particular areas and among them. There is no alternative for digital healthcare tools since an increasing number of consumers are using them, and interest in greater digital engagement continues to rise [64].

Based on the narrative literature review and the available data on e-health, we have distinguished and described the most important areas and factors in e-health implementation. Current studies indicate a diversified picture of e-health conditions in CEE countries. The purpose of our analysis was to investigate how these factors influenced (and to what extent) the implementation and use of e-health solutions within these countries.

Referring to the measurement of outcomes, and based on these indicators, the countries with high use of e-health services are as follows: Estonia (49 and 30%, respectively), Croatia (33 and 26%), Latvia (23 and 23%), and Slovenia (20 and 15%). Ordering prescriptions by telephone (or by emailing a primary physician) is considered a relatively simple procedure that patients very much appreciate. This is why it was used in the research.

We report the results of our QCA in more tables below. First, the positions of the analysed CEE countries are presented in Table 1, the countries we consider to be characterised by high use of e-health services in bold. Only Estonia was ranked above the EU27+3 average. Three other countries showed an average better than the EU27+3 average with respect to *Availability & Use Composite Indicators*. Six out of the 10 analysed CEE countries scored below the EU27+3 average with respect to both composite indicators. This shows that in the researched hospitals, the effective use of e-health tools and procedures is far below expectations (and, possibly, needs).

**Table 1:** E-health Composite Indicators for Hospitals in Selected Countries Compared to the EU27+3 Average (2012)

Country	Deployment Composite Indicators	Availability & Use Composite Indicators
**Estonia**	**+**	**+**
Croatia	–	+
Czech Republic	–	+
**Hungary**	**–**	**+**
Bulgaria	–	–
**Latvia**	**–**	**–**
Lithuania	–	–
Poland	–	–
Romania	–	–
**Slovenia**	**–**	**–**

Key: – means “below EU 27 + 3 average”; + means “above EU27+3 average”

Source: own compilation based on [66]

Similar research into acute hospitals [66] to a great extent strengthens the above-presented assessments. The selected findings from this study show that the use of e-health in CEE countries requires improvement.

Table 2 shows that the use of EMRs, EHRs and EPRs in four of the analysed countries (Croatia, Czech Republic, Estonia, and Hungary) is above the EU27+3 average. Furthermore, four of the countries (Croatia, Czech Republic, Estonia, and Latvia) showed better results than the EU27+3 average with respect to the exchange of clinical care information about patients. Nevertheless, in general, the positions of the national e-health profiles of the analysed countries within the EU27+3 are weak. This correlates with our findings.

**Table 2:** Type of Electronic Medical Records (EMRs) / Electronic Health Records (EHRs) / Electronic Patient Records (EPRs) and Exchange of Clinical Care Information Used by the Hospitals in Selected Countries

Country	Hospital-wide EMR/EHR/EPR shared by all clinical services (%)	Multiple loc/dept. EMR/EHR/EPR, which share information (%)	Multiple loc/dept. EMR/EHR/EPR, not sharing information (%)	No. EMR/EHR/EPR systems used in the hospital (%)	No. exchanges of clinical care information about patients (%)	Position of the national e-health profile within EU27+3
EU27+3 average	57	21	6	16	43	n/a
Czech Republic	82	3	3	12	35	exceeds
**Estonia**	**100**	**0**	**0**	**0**	**8**	**surpasses**
**Croatia**	**82**	**9**	**9**	**0**	**27**	**close**
Hungary	86	12	2	0	60	close
Latvia	75	12	12	1	26	uneven development
**Lithuania**	**23**	**30**	**10**	**37**	**75**	**behind**
Bulgaria	39	25	18	18	52	underperforming
Poland	50	7	7	36	71	behind
**Slovenia**	**33**	**17**	**17**	**33**	**83**	**behind**
Romania	61	17	4	18	71	significantly behind

Source: own compilation based on [66]

The results of the 2015 WHO GOE survey about e-health in CEE countries [32] present a chronology of the introduction of e-health policy in the CEE countries, as well as a juxtaposition of the introduction times, particularly of e-health national policies (Table 3). The first countries to introduce e-health policies were Estonia and Latvia, followed by Lithuania, Poland, Croatia, and Bulgaria. Table 4 contains the main observations derived from a comparison of the results for the CEE countries.

**Table 3:** The Introduction Years of E-health National Policies or Strategies in CEE Countries

Country	E-health	Universal health coverage	Health information system	Telehealth	Number of policies/strategies
Estonia	2003	2008	2014		3
Latvia	2005	2014			2
Lithuania	2010	2014		2011	3
Poland	2011	2004	2011		1
Croatia	2012	2012	2010	2010	4
Bulgaria	2014	1999			2
Czech Republic		2013	2002		2
Hungary		2014			1
Romania					–
Slovenia					–
Number of countries	6	8	4	2	

Source: own elaboration based on WHO’s Atlas of e-health country profiles [32]

**Table 4:** E-health in CEE Countries

E-health dimension	Main conclusions
1. E-health strategy	The most common e-health national policy or strategy in the CEE countries concerns universal health. Policy devoted to e-health can be observed in 6 of 10 CEE countries. E-health policies were established between 2003 and 2014. Policy for telehealth is less popular (two cases).
2. E-health funding sources	In all of the 10 countries, public sources fund e-health. The next popular source is donor/non-public funding.
3. Training in e-health	E-health capacity building exists in each of the countries investigated, with a slight dominance of pre-service training.
4. Legal frameworks for e-health	The most multipurpose e-health policies exist in Estonia (12), Lithuania (12), Latvia (11), next Slovenia (10), Hungary (9), Romania (9), Bulgaria (8), and finally Poland (5), Croatia (4) and Czech Republic (4).
5. Telehealth	Only in three countries have telehealth programmes been established at a national level.
6. Electronic health records (EHRs)	Countries that reported possessing a national EHR system also reported having adequate legislation regulating its use. The system appears in three countries (Estonia, Lithuania, and Romania).
7. mHealth programmes country overview	mHealth programmes were most intensively applied in Estonia and Lithuania (8 of 14), followed by Latvia (7/14) and Hungary (4/14).
8 Social media	Usage not reported
9. Big data	Only in two countries is this issue considered: Lithuania and Slovenia.

Source: own elaboration based on WHO’s Atlas of e-health country profiles [32]

Table 5 lists the final set of conditions for the implementation of e-health that we used for our analysis. All these conditions are already mentioned in the eHealth survey by the WHO Global Observatory [31, 32].

**Table 5:** Conditions for the Implementation of E-health

Category	Abbreviation	Conditions	Data treatment explanation	Codes
National policy or strategy	POLICY	National e-health policy	Reported policy for particular country.	0 – absent 1 – present
	HIS	National health information system (HIS) policy or strategy	Reported policy for particular country.	0 – absent 1 – present
Funding sources	FUNDING	E-health funding sources	Covers several sources: public funding; private or commercial funding; donor/ not-public funding; public-private partnership.	0 – no or one funding source reported 1 – three funding sources reported
Capacity building	TRAINING	E-health capacity building	Includes: Health sciences students – Pre-service training in e-health and health professionals – In-service training in e-health.	0 – no or only pre-service or only in-service training 1 – both pre- and in-service training present
Legal frameworks	MED_JURIS	Policy of e-health legislation defines medical jurisdiction, liability or reimbursement of e-health services	Reported purpose for given country.	0 – absent 1 – present
	DATASHARE	Policy of e-health legislation governs the sharing of digital data between health professionals in other health services in the same country through the use of an her	Reported purpose for given country.	0 – absent 1 – present
	IND_ACC	Policy of e-health legislation allows individuals electronic access to health-related data (when held in EHRs)	Reported purpose for given country.	0 – absent 1 – present
Programmes at national level	TELEPROG	Telehealth	Includes five telehealth programmes: teleradiology, teledermatology, telepathology, telepsychiatry, and remote patient monitoring. We included only established or pilot programmes at the national level.	0 – absent 1 – present
	HER	Electronic Health Records (EHRs)	We included only the national EHR system	0 – absent 1 – present
	MHEALTH	mHealth programmes country overview	We counted only those at the national level that are in the pilot or established phase.	0 – absent 1 – present

Referring to the QCA analysis, we conducted conventional crisp set analysis, where set membership is binary (Table 6). Our data also indicate that there are three combinations of conditions leading to the effective implementation of e-health services (Table 7). The most complex situation that we observe is found in combination 3. Nine conditions led to the high use of e-health services in Estonia. The other two combinations possess fewer conditions: only TRAINING and MHEALTH are found in both.

**Table 6:** Data set

	OUTCOME	CONDITIONS									
Country	Use of e-health services	National e-health policy or strategy	National health information system (HIS) policy	E-health funding sources	Pre- and in-service training	Policy of e-health legislation defines medical jurisdiction, liability or reimbursement of e-health services	Policy of e-health legislation governs the sharing of digital data	Policy of e-health legislation allows individuals electronic access to health-related data	Telehealth established programme	National EHR system	mHealth programmes
COUNTRYID	USE	POLICY	HIS	FUNDING	TRAINING	MED_JURIS	DATASHARE	IND_ACC	TELEPROG	EHR	MHEALTH
Bulgaria	0	1	0	0	1	0	0	1	0	0	0
Croatia	1	1	1	0	1	0	0	0	1	0	0
Czech Rep.	0	0	1	0	0	0	0	0	0	0	0
Estonia	1	1	1	1	1	0	1	1	1	1	1
Hungary	0	0	0	0	0	0	1	1	0	0	1
Latvia	1	1	0	0	1	1	1	1	0	0	1
Lithuania	0	1	0	1	1	0	0	1	1	1	1
Poland	0	1	1	0	1	0	0	0	0	0	0
Romania	0	0	0	0	0	1	1	1	0	1	0
Slovenia	1	0	0	0	1	1	0	1	0	0	1

**Table 7:** Results of the analytical procedure

Condition	Combination 1	Combination 2	Combination 3
POLICY	×	●	●
HIS	○	●	●
FUNDING	○	○	●
TRAINING	●	●	●
MED_JURIS	●	○	○
DATASHARE	○	○	●
IND_ACC	●	○	●
TELEPROG	○	●	●
HER	○	○	●
MHEALTH	●	●	●
COUNTRYID	LV, SLO	HU	EST

Key: ● – presence, ○ – absence, × − not included

Combination 1 is found in Latvia and Slovenia, while combination 2 is found in Hungary. Then, we searched for parsimonious solutions, which refer to any causal combination that uses at least some of the causal conditions specified in the complex solution as a valid solution of the truth table, as long as it contains all of the causal conditions specified in the parsimonious solution. Nine such solutions appeared. In Estonia and Hungary, national e-health information system (HIS) policy and the established telehealth programme (TELEPROG) are crucial conditions for achieving the outcome. In Latvia and Slovenia, eight parsimonious combinations emerged. In two of them, the key conditions are related to both established m-health programmes and pre- and in-service training.

In the next four combinations, the condition related to legally defined medical jurisdiction, liability or reimbursement of e-health services (MED_JURIS) was found. This condition is also present in the next combination, along with established m-health programmes (although a national information system or policy is not reported). In the last parsimonious combination, established m-health programmes appear, but the sharing of digital data between health professionals in other health services in the same country via EHR is not regulated.

We also wanted to know which conditions are necessary for the successful outcome to occur. For this purpose, we examined the necessary conditions via the consistency and coverage scores for individual conditions (Table 8). Consistency indicates the degree to which the causal condition is a superset of the outcome, and coverage indicates the empirical relevance of a consistent superset. There is one condition that is always associated with a successful outcome: pre- and in-service training. This finding supports a previous study on implementing e-health initiatives, where such initiatives would succeed if, among others, they fit well with the skill sets of the existing staff [34]. The next three necessary conditions with a consistency of 0.75 are as follows: POLICY, IND_ACC, and MHEALTH. They can be explained as the possibility of individual access to their data within m-health programmes guaranteed by national e-health policy.

**Table 8:** Analysis of necessary conditions

Condition	Consistency	Coverage
TRAINING	1.00	0.57
POLICY	0.75	0.50
IND_ACC	0.75	0.43
MHEALTH	0.75	0.61
HIS	0.50	0.50
MED_JURIS	0.50	0.68
TELEPROG	0.50	0.68
FUNDING	0.25	0.50
DATASHARE	0.25	0.25
her	0.25	0.33

After defining the set of conditions leading to effective e-health implementation, we wanted to investigate which conditions (or lack of conditions) contribute to a lack of high use of e-health services. The analysis showed that none of the countries characterised by low use of e-health services have the same combination of conditions (Table 9).

**Table 9:** Results of the analytical procedure

Condition	Combination 1	Combination 2	Combination 3	Combination 4	Combination 5	Combination 6
POLICY	○	○	○	●	●	●
HIS	●	○	○	●	○	○
FUNDING	○	○	○	○	○	●
TRAINING	○	○	○	●	●	●
MED_JURIS	○	●	○	○	○	○
DATASHARE	○	●	●	○	○	●
IND_ACC	○	●	●	○	●	●
TELEPROG	○	○	○	○	○	●
HER	○	○	○	○	○	●
MHEALTH	○	○	●	○	○	●
COUNTRYID	CZ	RO	HA	PL	BG	LT

Key: ● means “condition present”; ○ means “condition absent”

We also generated four parsimonious solutions. In the first solution, low e-health service use is connected with a lack of established TELEPROG and MHEALTH programmes. In the second, the lack of both established telehealth programmes and legally defined medical jurisdiction, liability or reimbursement of e-health services occur. The third is related to the lack of a national information system and legally defined medical jurisdiction, liability or reimbursement of e-health services. The last includes the cases in which the sharing of digital data between health professionals in other health services in the same country through the use of an HER is regulated, but a national information system is missing.

## Discussion

As indicated above, the findings are inconclusive, although the analysed countries have the same heritage (post-socialist political systems with a centralised Semashko’s model of healthcare [19]) and a rather homogenous cultural profile. Our analysis shows that there is no one way that leads to the successful or unsuccessful implementation of e-health. However, for both implementation outcomes, some factors are important (e.g., TRAINING in regard to success or MED_JURIS, TELEPROG or HIS in regard to failure). In other words, boundary conditions for the successful implementation of e-health are MED_JURIS, TELEPROG and HIS, while TRAINING is a necessary condition.

Our findings correspond to the results of other studies. Sabes-Figuera & Maghiros [66] researched hospitals in 27 EU countries (plus three others: Iceland, Norway and Croatia) between 2010 and 2012 using composite indicators grouped as *e-health deployment* and *e-health availability and use*. The indicator for *e-health deployment* was based on 45 variables grouped into four dimensions (infrastructure, applications, health information exchange, and security and privacy). The *e-health availability and use* indicator was based on information from a survey on the level of availability and use in each hospital of 39 different e-health functionalities pertaining to four categories (view/input information on EHR, clinical decision support on EHR, health information exchange, and telehealth) [33].

A separate but important issue is the level of e-health skills, first among patients (both current and prospective). Vicente & Madden [67] prove that e-health skills can be tied not only to level of education (as shown through WHO data) but also to age and health status. Their findings show that the most vulnerable groups comprise — along with the less educated — the sick and the elderly [67]. The lack of e-health skills among senior citizens is broadly recognised [51]. This fact gains value when keeping in mind that e-health strategies have enormous potential to support active ageing [68].

Our findings are consistent with the general mechanisms/rules for managing change in healthcare as indicated in the literature [69, 70] and with the conditions for more specific, medically-oriented changes introduced in healthcare organisations [71].

This study has certain limitations that can be classified as internal or external. The first relates to the character of the WHO data on e-health in the analysed CEE countries. Notwithstanding the value of the data, one may observe certain weaknesses. As we used secondary data from WHO survey, the empirical results reported in this study should be considered in the light of limitations related to experts’ evaluation of given dimension of e-health policy for analysed country. More rigorous analysis of the WHO data shows that some links on the WHO web page on national strategies on e-health [31] were either not present or not found and that the indicated acts and legal regulations were available exclusively in local languages.^1^ One should also point at the focus on macro level and programmes initiated and run by the government leaving various interesting regional and local initiatives and programmes apart. Such an unwilling bias does not mean that the analysis is valid only for countries having comparatively highly centralized health care systems. Everywhere in the world, highly decentralized and market-oriented national health care systems including, the government is responsible for nationwide health policy. There are not systemic barriers for the government to initiate, support and run initiatives and programmes aimed at e-health.

Another important issue is the slightly biased, positive manner in which national strategies of e-health development are presented.^2^ Separate but worth acknowledgement is the fact that the WHO data do not cover the most recent developments (i.e., 2014–2018) of e-health in the respective countries.

Last but not least is the size (only 10 countries) and composition (post-socialist, CEE countries) of the sample thus one may indicate that the findings have limited value.

Moreover, a number of challenges are associated with the QCA method. The data we used and their weaknesses directly affect the analyses. They determined the outcome and conditions. From this perspective, identifying the conditions was a great challenge. The selection process had to be restrictive, as QCA does not allow the inclusion of a large number of conditions. Another challenge emerged concerning the way in which we assessed the outcomes. As noted, we defined the outcome as the successful implementation of e-health services. However, other studies on healthcare in selected Central Eastern European countries used the overall satisfaction of healthcare users [72]. The implementation of the e-health strategy is a very complex process. Our analyses are related only to the paths of e-health implementation in CEE countries thus consequently the findings and conclusions cannot be directly applied to other countries or sub-continents characterized by significant technological, economic, social, demographic differences. Therefore, an interesting subject of study would be the comparison of the implementation paths in CEE and other countries. The external limitations of this study are related the absence of a broader context of e-health development, including the development of ICT infrastructure and ICT literacy. Here, the functional side of e-health in the respective countries should be rigorously analysed with a special emphasis on medical professionals’ and patients’ skills in e-health since the lack of ICT skills negatively influences regional e-health implementation [65].

## Conclusions

The results of the study provide various conclusions and implications. First, we observe that analysed CEE countries are slowly progressing with e-health implementation, with Estonia being the undisputed leader. The selected indicators of EMRs, EHRs and EPRs and an exchange of clinical care information in hospitals show that the analysed countries are trying to catch up with the EU27+3 average; in general, however, the positions of the national e-health systems in the analysed countries within the EU27+3 remain weak. The most advanced countries in e-health service use are Latvia, Slovenia, Hungary, and Estonia. All of them (except for Slovenia) are pioneers in introducing e-health policy in the CEE countries.

Slovenia is an atypical case according to our analysis. This country reported relatively high use of e-health services and was characterised by a lower number of conditions facilitating the use of e-health, which is consistent with the findings of Sabes-Figuera & Maghiros [66] and our analysis. An additional query on e-health development in Slovenia shows that an e-health project was launched in 2005 but that it is still not fully operational (expected in 2020) due to various deficiencies [73]. The QCA analysis used for e-health development in the analysed CEE countries showed that to achieve success in e-health implementation, legal medical jurisdiction, teleprogrammes and EHRs must be in place (supplemented by adequate training). Moreover, the paper indicates a considerable research gap with respect to e-health implementation in the analysed countries.

Referring to managerial and policy implications, the paper shows that the use of e-health services depends on enabling technology-supporting relations between patients and well-skilled clinicians. This study fosters observation regarding the need “for hospitals and decision makers to clearly identify and act on the drivers of successful implementations [of e-health solutions]” [74]. Moreover, it reduces uncertainty about the factors having an influence, as many barriers are observed [34].

For policymakers, it is clear that governments should support the development of e-health programmes via e-health legislation with special attention to medical jurisdiction, liability or reimbursement of e-health services. This study observes several obstacles to introducing m-health policy, such as data security, licensure, and patient confidentiality and privacy [75]. Another recommendation for managers and policy-makers is the development of training programmes for health science students and health professionals. This is the condition sine qua non for spreading the use of e-health services in countries where these solutions are not popular among society.

## Data Availability

The datasets used and analysed during the current study are available from the corresponding author on reasonable request.

## Abbreviations

CEE: Central Eastern European
EHRs: Electronic Health Records
EMRs: Electronic Medical Records
EPRs: Electronic Patient Records
EU: European Union
EU27+3: European Union 27 countries plus three others: Iceland, Norway and Croatia
HIS: Health information system
HIT: Health IT
ICT: Information and Communication Technology
QCA: Qualitative Comparative Analysis
WHO GOE: WHO Global Observatory for eHealth

## Footnotes

1 For example, the source for the Hungarian Information Society Strategy, Health and Social Services was removed (http://www.eski.hu/eprogram/english/Mitsesz.pdf). The Lithuanian eHealth strategy is available only as *Dėl Lietuvos E. Sveikatos 2007–2015 Metų Plėtros Strategijos Patvirtinimo* (http://www3.lrs.lt/pls/inter3/oldsearch.getfmt?C1=e&C2=306637), and the Romanian strategy is available as *Romania Strategia de e-Sănătate a Ministerului Sănătăţii* (http://www.ms.gov.ro/documente/256_588_Anexa%20strategie%20e-sanatate.doc).

2 Since there were national governments and/or ministries of health responsible for the preparation of such strategies, it is not surprising that they were presented in a positive and optimistic way.
